# Supplementary material for: From sequence to enzyme mechanism using multi-label machine learning
Source: BMC Bioinformatics. 2014 May 19;15:150. doi: 10.1186/1471-2105-15-150 (PMC4229970; doi:10.1186/1471-2105-15-150)
Supplement: Additional file 2 — Java code of ml2db. Additional file ml2db_code.tar.gz contains the Java source code to run the multi-label machine learning experiments and save the results to database. The code’s Javadoc is included. [file 1471-2105-15-150-S2.zip › additional file 2/ml2db/ml2db/doc/main/ml2db/mulan/measuremetrics/package-use.html]

Uses of Package main.ml2db.mulan.measuremetrics


JavaScript is disabled on your browser.


- Overview
- Package
- Class
- Use
- Tree
- Deprecated
- Index
- Help

- Prev
- Next

- Frames
- No Frames

- All Classes

# Uses of Package main.ml2db.mulan.measuremetrics

- Packages that use main.ml2db.mulan.measuremetrics

  | Package | Description |
  | --- | --- |
  |  |  |
  | --- | --- |
  | main.ml2db.mulan.measuremetrics |  |
  | test.ml2db.mulan.measuremetrics |  |
- Classes in main.ml2db.mulan.measuremetrics used by main.ml2db.mulan.measuremetrics

  Class and Description

  AnnotationsReader

  Two sets of [instance, annotation] couples are taken as input, one containing
  "predictions" and another containing the "truth" .
- Classes in main.ml2db.mulan.measuremetrics used by test.ml2db.mulan.measuremetrics

  Class and Description

  AnnotationsReader

  Two sets of [instance, annotation] couples are taken as input, one containing
  "predictions" and another containing the "truth" .

  PredictionsPerformanceMeasurer

  Generates prediction metrics by comparing 1.

- Overview
- Package
- Class
- Use
- Tree
- Deprecated
- Index
- Help

- Prev
- Next

- Frames
- No Frames

- All Classes
